# Exploring Phenotypes for Disease Resilience in Pigs Using Complete Blood Count Data From a Natural Disease Challenge Model

**DOI:** 10.3389/fgene.2020.00216

**Published:** 2020-03-13

**Authors:** Xuechun Bai, Austin M. Putz, Zhiquan Wang, Frédéric Fortin, John C. S. Harding, Michael K. Dyck, Jack C. M. Dekkers, Catherine J. Field, Graham S. Plastow, PigGen Canada

**Affiliations:** ^1^Livestock Gentec, Department of Agricultural, Food and Nutritional Science, University of Alberta, Edmonton, AB, Canada; ^2^Department of Animal Science, Iowa State University, Ames, IA, United States; ^3^Centre de Développement du Porc du Québec, Inc., Quebec City, QC, Canada; ^4^Department of Large Animal Clinical Sciences, University of Saskatchewan, Saskatoon, SK, Canada

**Keywords:** natural disease challenge model, disease resilience, complete blood count, genetic parameters, pigs

## Abstract

Disease resilience is a valuable trait to help manage infectious diseases in livestock. It is anticipated that improved disease resilience will sustainably increase production efficiency, as resilient animals maintain their performance in the face of infection. The objective of this study was to identify phenotypes related to disease resilience using complete blood count (CBC) data from a wean-to-finish natural disease challenge model, established to mimic the disease pressure caused by many common pathogens at the commercial level of pig production. In total, 2433 F1 crossbred (Landrace × Yorkshire) barrows that went through the natural disease challenge model were classified into four groups (resilient, average, susceptible, and dead) based on their divergent responses in terms of growth and individual treatment. Three sets of blood samples for CBC analysis were drawn at 2-weeks before, and at 2- and 6-weeks after the challenge: Blood 1, Blood 3, and Blood 4 respectively. CBC of Blood 1 taken from healthy pigs before challenge did not show differences between groups. However, resilient animals were found to be primed to initiate a faster adaptive immune response and recover earlier following infection, with greater increases of lymphocyte concentration from Blood 1 to Blood 3 and for hemoglobin concentration and hematocrit from Blood 3 to Blood 4, but a lower neutrophil concentration from Blood 3 to Blood 4 than in susceptible and dead animals (*FDR* < 0.05). The CBC traits in response to the challenge were found to be heritable and genetically correlated with growth and treatment, which may indicate the potential for developing CBC under disease or commercial conditions as a phenotype in commercial systems as part of developing predictions for disease resilience.

## Introduction

Disease resilience is defined as an animal’s ability to maintain a relatively undepressed performance in the face of infection (Albers et al., 1987; Mulder and Rashidi, 2017). In pig breeding, disease resistance, which is defined as the ability to suppress establishment and subsequent development of infection, has been generally discussed in terms of making genetic improvement of herd health (Albers et al., 1987; Bishop and Stear, 2003; Guy et al., 2012). For example, the discovery of a polymorphism at bp 307 (G/A) in the fucosyltransferase gene (*FUT1*) associated with susceptibility/resistance to infection with F18 fimbriated *Escherichia coli* (ECF18) made it possible to select for ECF18 resistant pigs (Meijerink et al., 1997, 2000). Pigs that are homozygous for the resistant allele are resistant to ECF18 due to the non-adhesion of ECF18 in the small intestine (Meijerink et al., 1997; Bao et al., 2012). However, such complete resistance to a pathogen is not common, and selection for resistance to a specific pathogen may have unfavorable consequences for other production traits (Wilkie and Mallard, 1999; Guy et al., 2012). Currently, the challenge of infectious diseases in the pig industry is that a multitude of pathogens exists around the world (Zimmerman et al., 2012). Some pathogens, including porcine reproductive and respiratory syndrome virus (PRRSV), can also modulate the immune system to increase susceptibility to other pathogens while suppressing the immunologic memory of the host for the same pathogen (Zhu et al., 2010). Therefore, selective breeding for resilient animals that can maintain a relatively undepressed performance in a commercial system that typically harbors abundant infectious agents could be a pragmatic way to help maintain or even improve the productivity of the swine industry.

**FIGURE 1:**
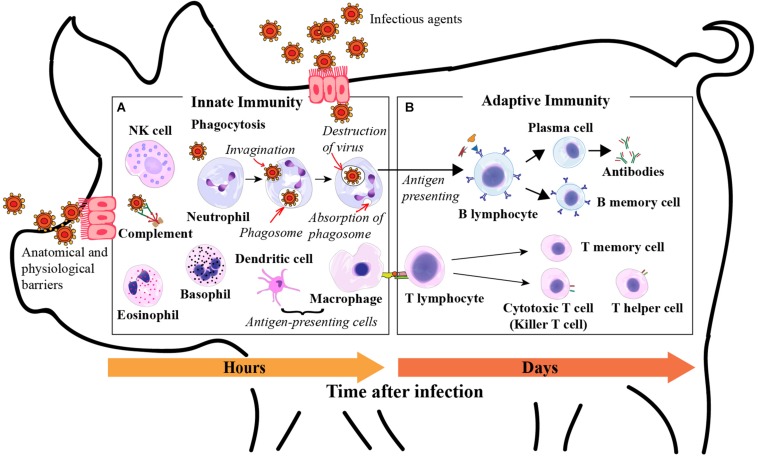
Roles of white blood cells in innate immunity. **(A)** Phagocytosis is the process by which phagocytic cells recognize and ingest microbes for intracellular killing. Phagocytes include neutrophils, monocytes, dendritic cells, and eosinophils; Neutrophils, eosinophils, and basophils are granulocytes, the granules present in their cytoplasm contain biochemical mediators that serve inflammatory and immune functions; Eosinophils and basophils combat parasites through production of toxic proteins and histamine respectively; Dendritic cells produce cytokines that recruit white blood cells and initiate adaptive immune responses, and also present antigens to the adaptive immune system; Natural killer (NK) cells are a class of lymphocytes that recognize and kill infected cells to stop the spread of an infection; The complement system consists of a set of plasma proteins that act together to defend against extracellular pathogens. Roles of white blood cells in adaptive immunity. **(B)** B lymphocytes mediate humoral immunity by secreting antibodies into the circulation and mucosal fluid to neutralize and eliminate extracellular infectious agents; T lymphocytes characterize cell-mediated immunity and kill host cells that are harboring infectious agents in the cytoplasm. Derived from Janeway et al. (2001), Abbas et al. (2015), and Elsevier Health Sciences and Khan Academy (2019).

Direct selection for disease resilience is generally not feasible, because it is impractical to obtain heritable measures of resilience in the high health nucleus herds where the selection of elite breeding animals takes place (Wilkie and Mallard, 1999). Moreover, it is also challenging to appropriately characterize resilience because it is a complex trait composed of multiple biological functions, such as production, health, nutrient status, and other dynamic elements, including the efficiency of immune response and the rate of recovery from infection (Friggens et al., 2017). Many studies have explored the relationship of immune traits with performance. These include the use of white blood cell traits (Figure 1), which are reported to be moderately to highly heritable and genetically correlated with an animal’s performance (Henryon et al., 2006; Clapperton et al., 2008, 2009; Flori et al., 2011; Mpetile et al., 2015). In addition to white blood cells, red blood cells and platelets have also been shown to play multiple roles in the immune system to help defend against pathogens, and these also have the potential to be genetically correlated with an animal’s performance (Gershon, 1997; Liepke et al., 2003; Jiang et al., 2007; Rondina and Garraud, 2014; Hottz et al., 2018). Complete blood count (CBC) is a clinical measure used to evaluate the concentration and relative proportion of circulating blood cells and may be a practical measure of immune response and, therefore, could be a candidate phenotype for disease resilience. Moreover, CBC also evaluates the volume and concentration of red blood cells and hemoglobin to provide information about oxygen-carrying capacity and anemia, which are of concern during the disease process, with further impacts on animal performance (George-Gay and Parker, 2003).

Therefore, the objectives of this study were: (1) to assess CBC profiles of pigs that exhibited divergent performance in terms of growth and individual treatment in response to a polymicrobial infectious challenge; and (2) to estimate heritabilities of CBC traits and genetic correlations of CBC with growth and treatment rates following the disease challenge.

## Materials and Methods

This study was carried out in accordance with the Canadian Council on Animal Care guidelines (CCAC^1^). The protocol was approved by the Animal Protection Committee of the Centre de Recherche en Sciences Animales de Deschambault (15PO283) and the Animal Care and Use Committee at the University of Alberta (AUP00002227). The project was fully overseen by the Centre de Développement du Porc du Québec (CDPQ) and the herd veterinarian together with project veterinarians.

### Natural Disease Challenge Model and Data Collection

A natural disease challenge model was established for wean-to-finish pigs at Deschambault, in the province of Québec, Canada. There were two main facilities in the model: (1) a healthy quarantine unit providing a 3-week nursery after weaning, and (2) a test station that consisted of a 4-week late nursery stage (40 to 68 days of age on average) and a grow-to-finish stage for approximately 16 weeks (69 to 181 days of age on average). The number of pigs per pen was approximately 4, 7, and 13 for the healthy quarantine unit, the test station late nursery, and the test station grow-to-finisher, respectively. Pigs were first exposed to the challenge in the test station in the late nursery, which aimed to represent and simulate a severe disease pressure caused by multiple pathogens found at the commercial level of production to maximize the expression of phenotypic and genetic differences associated with resilience. The test station barn was operated as a high health status facility prior to the introduction of the disease agents. Common disease-causing pathogens found in commercial farms were established by co-introducing commercial seeder pigs with known diseases with the first four batches of healthy pigs, including two viruses (three different strains of PRRSV and two strains of swine influenza A virus), five bacterial pathogens (*Mycoplasma hyopneumoniae*, *Haemophilus parasuis*, *Brachyspira hampsonii*, *Salmonella enterica* serovar *typhimurium*, and *Streptococcus suis*), and two parasites (*Cystoisospora suis* and *Ascaris suum*). For the data used in this study, every batch was confirmed to have been exposed to PRRSV in the test station based on randomly sampling of blood from a subset of individuals for RT-PCR 4 weeks post-challenge and enzyme-linked immunosorbent assay (ELISA) 6 weeks post-challenge. In addition to the introduced pathogens, other multiple disease-causing pathogens were also identified in the challenge facility, including porcine circovirus type-2 (PCV2), porcine rotavirus A, *Erysipelothrix rhusiopathiae*, *Staphylococcus hyicus*, and some undefined minor pathogens. However, these were not necessarily identified in all batches, as disease pressure varied by batch and on a seasonal basis. Not all pigs were exposed to all pathogens, as would be the case on a commercial farm. The level of mortality in a batch was carefully monitored and adjustments made to address the situation for reasons of animal ethics. For example, if the average mortality rate of each batch was more than 8% during the nursery stage, then group medication was applied through water and feed on a batch-level. If the challenge based on previous data was deemed to be too severe then direct nose-to-nose contact between batches in the challenge nursery was stopped.

**FIGURE 2:**
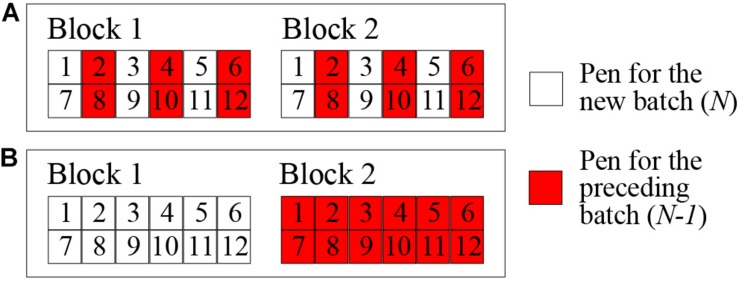
Pen arrangements in the test station late nursery for the nose-to-nose direct challenge **(A)** and for the indirect contact during the period of excessively high pressure of challenge **(B)**.

Healthy F1 crossbred (Landrace × Yorkshire) castrated male weaned pigs were provided in rotation by seven genetic suppliers, all members of PigGen Canada. A total of 2743 pigs were introduced in 42 batches at 3-week intervals. Each batch consisted of approximately 65 or 75 pigs from one of the genetic suppliers. Every seven batches constituted a cycle. All weaned pigs arrived at an average age of 21 days old and were housed in a healthy quarantine unit, representing a 3-week nursery stage. For the first cycle, the quarantine unit and test station were in the same building connected by a hallway, but strict biosecurity protocols were practiced between them. Since the biosecurity practices were insufficient to stop the spread of pathogens from the test station to the clean quarantine, a separate quarantine unit located approximately 1 km south of the test station was set up for cycles 2 to 6 and kept free of disease by adhering to strict biosecurity protocols. Every 3 weeks, a new batch of approximately 40-day-old pigs was transferred from the quarantine nursery to the test station late nursery and exposed to the disease challenge by direct nose-to-nose contact with the preceding batch for 1 week (Figure 2A). The challenge was set up as a continuous flow system in order to maintain a steady disease challenge without repeatedly introducing commercial pigs and pathogens. During periods of very high challenge pressure, as identified by rates of morbidity and mortality, batches (*n* = 12) were not challenged by direct nose-to-nose contact. Pigs in these batches were allocated to nursery pens physically separated from the preceding batch (Figure 2B) to help maintain the mortality rate below the target level established by the Animal Protection Committee.

The first blood sample for CBC (Blood 1) was collected on all pigs in the quarantine nursery at an average age of 26 days, 5 days post-arrival from their farm of origin. Two weeks after the first sampling, pigs were transferred to the test station and naturally exposed to multiple pathogens from 40 to approximately 181 days of age, when they reached the target slaughter weight of 130 kg. Blood 2 was collected immediately before transferring to the test station nursery at 40 days of age to measure antibody-mediated responses for a separate study of the immune response. No CBC was obtained on these Blood 2 samples. The second CBC blood sample (Blood 3) was collected on all pigs 2-weeks after transferring to the test station, at an average age of 54 days. The third CBC blood sample (Blood 4) was collected at approximately 82 days of age, 4 weeks after the collection of Blood 3, and 6 weeks after the transfer to the test station.

All blood samples were taken from the jugular vein, and the samples for CBC were collected into K2 ethylenediaminetetraacetic acid (EDTA) tubes (BD Vacutainer^®^ blood collection tubes, New Jersey, United States). Samples were shipped overnight with ice packs and received by the University of Alberta for the CBC analysis using the ADVIA^®^ 2120i Hematology System (Siemens Healthineers, Erlangen, Germany) within 24 to 48 h.

Body weights of each pig were measured every 3 weeks. Mortality and morbidity of each batch, as well as the reasons for death were also recorded on an individual pig basis. All medical treatments were recorded, including individual medication given on a case-by-case basis throughout the lifetime of the pig, as well as group treatments that were given on a batch-level. Of note, due to significant problems in managing the associated impact caused by PCV2 in cycle 1, Ingelvac CircoFLEX^®^ PCV2 vaccination (Boehringer Ingelheim, Ingelheim am Rhein, Germany) was administered intramuscularly as per the label instructions to pigs before entering the test station from the second cycle onwards.

### Genotyping

The genotyping of animals was performed at Delta Genomics (Edmonton, AB, Canada) using the 650K Affymetrix Axiom^®^ Porcine Genotyping Array. In total, 658,692 single nucleotide polymorphisms (SNPs) were included on the chip. Raw Affymetrix SNP data for each cycle were processed separately at Delta Genomics with the Axiom Analysis Suite, using all defaults. Missing genotypes were imputed using FImpute (Sargolzaei et al., 2014). Sscrofa 11.1 was used as the reference genome. Quality control was performed using the preGSf90 software from the BLUPF90 family of programs to remove SNPs with a minor allele frequency lower than 0.01 and call rates lower than 0.90. Overall, genotypes for 2593 animals from all six cycles were used, with 475,839 SNPs remaining after processing and quality control.

### Traits

The CBC traits used for this study were grouped into three categories: (1) six white blood cell traits, including total white blood cell concentration (WBC,  10^3^/μL), neutrophil concentration (NEU,  10^3^/μL), lymphocyte concentration (LYM,  10^3^/μL), monocyte concentration (MONO,  10^3^/μL), eosinophil concentration (EOS,  10^3^/μL), and basophil concentration (BASO,  10^3^/μL); (2) seven red blood cell traits, consisting of red blood cell concentration (RBC,  10^6^/μL), hemoglobin concentration (HGB,  g/L), hematocrit (HCT,  %), which measures the volume percentage of packed red blood cells in blood, mean corpuscular volume (MCV,  fL), mean corpuscular hemoglobin (MCH,  pg), mean corpuscular hemoglobin concentration (MCHC,  g/L), and red blood cell distribution width (RDW,  %), which evaluates the variability in size of red blood cells; and (3) two platelet traits, including platelet concentration (PLT,  10^3^/μL) and mean platelet volume (MPV, fL). In addition to these measurable traits for each blood sample, changes of CBC traits between blood samples collected at different time points were also calculated for each animal, which will be referred to as Δ13 for the change from Blood 1 to Blood 3 (Blood3 – Blood1), Δ34 for the change from Blood 3 to Blood 4 (Blood4 – Blood3), and Δ14 for the change from Blood 1 to Blood 4 (Blood4 – Blood1).

The growth rate of each animal in the grow-to-finish phase (GFGR) was estimated using linear regression of body weights collected from an average of 69 days old to the endpoint, i.e., when the pig died or when it reached the target slaughter weight at approximately 181 days old. The GFGR for animals that died before reaching the grow-to-finish stage was set to missing in the analyses. Treatment rate (TR) for each animal was the number of treatment events in the natural challenge barn, standardized by the number of days spent in the natural challenge barn (TR = number of treatment events/days × 100%). Group treatments given on the batch-level were not included because these would be accounted for in the model by fitting the fixed effect of batch. The TR for animals that died before receiving any treatment was set to missing.

### Classification of Pigs Based on Resilience

**FIGURE 3:**
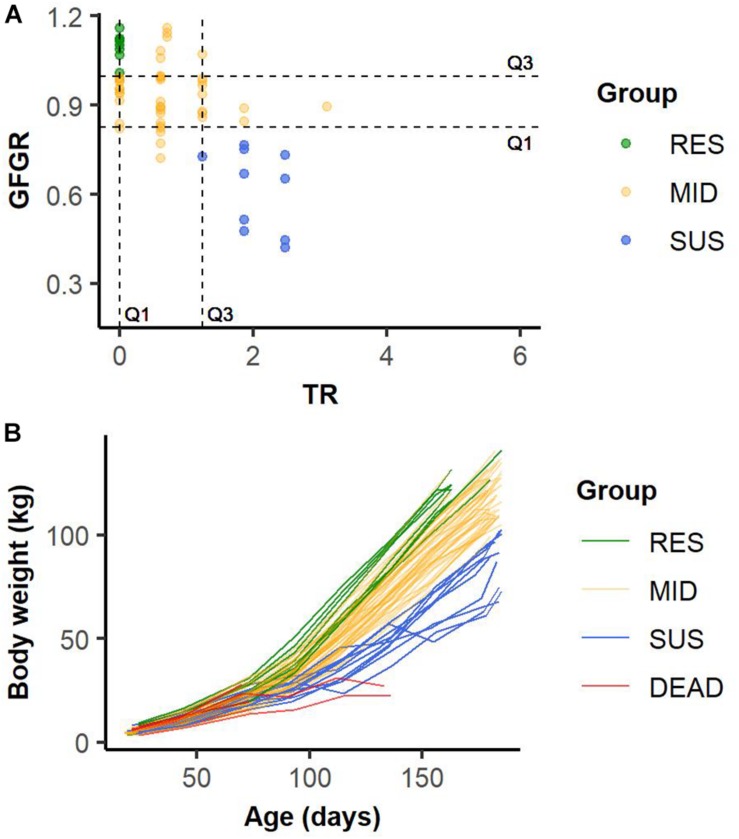
Example of the classification of slaughtered animals into resilient (RES), average (MID), and susceptible (SUS) groups based on the first (Q1) and the third (Q3) quartiles of grow-to-finish growth rate (GFGR) and treatment rate (TR) in Batch 14 **(A)**. Example of growth curves for animals in resilient (RES), average (MID), susceptible (SUS) and dead (DEAD) groups in Batch 14 **(B)**.

Based on resilience indicated by phenotypes of GFGR and TR, pigs were classified into four groups as “resilient (RES),” “average (MID),” “susceptible (SUS),” and “dead (DEAD)” by batch. Within each batch, slaughtered pigs that had equal or higher GFGR than the third quartile (Q3, 75% quartile), and equal or lower TR than the first quartile (Q1, 25% quartile) of all slaughtered pigs in the batch were classified as RES; slaughtered pigs that had equal or lower GFGR than the Q1 and equal or higher TR than the Q3 of all slaughtered pigs in the batch were regarded as SUS; the rest of the slaughtered animals, which had moderate TR and GFGR, were classified as MID (Figure 3). The influence caused by the environmental changes and differences among batches were controlled and minimized by classifying animals within each batch. Among 2593 genotyped pigs, mortalities (*n* = 160) caused by hernia, fighting, fracture, sampling, or sudden death due to unclear reasons were excluded from the analysis. Of the remaining 2433 pigs, 505 (21%) pigs that died as a result of infectious disease were classified as DEAD. For the 1928 pigs that were slaughtered at market body weight in the six cycles, 213 (9%) pigs were in the RES group, 1505 (61%) pigs were in the MID group, and 210 (9%) pigs were in the SUS group.

### Statistical Analyses

#### Removal of Outliers

Due to the relative complexity of the sample handling, shipping conditions, and laboratory analysis, outliers for the measures of CBC traits could be the result of damaged samples with hematological issues including hemolysis and clotting, or mechanical problems of the Hematology System used to measure CBC from blood samples. Such outliers were detected and removed using the Adjusted Boxplot in R (R Core Team, 2017, Package ‘robustbase’). It is a robust measure of skewness in the determination of thresholds for the removal of outliers and can avoid erroneously declaring points as outliers in a skewed distribution (Hubert and Vandervieren, 2008). The skewness of a CBC trait was measured using Medcouple (Brys et al., 2004). Thresholds for removing outliers for CBC measures were determined by several parameters, including Medcouple (MC), first quartile (Q1), third quartile (Q3), and interquartile range between Q1 and Q3 (IQR). The lower and upper bounds for a right-skewed distribution (MC > 0) were Q1 − 1.5^(–4MC)^ × IQR and Q3 + 1.5^(3MC)^ × IQR; for a left-skewed distribution (MC < 0), the lower and upper bounds were Q1 − 1.5^(–3MC)^ × IQR and Q3 + 1.5^(4MC)^ × IQR; and for a symmetric distribution (MC = 0), the outliers were removed using Tukey’s boxplot (lower bound Q1 − 1.5 × IQR, upper bound: Q3 + 1.5 × IQR) (Seo, 2006; Hubert and Vandervieren, 2008). All CBC measures outside of the upper and lower bounds were removed as outliers.

#### Models

The likelihood ratio test in ASReml 4.1 was used to determine the significance of different environmental random terms for litter and pen effects by comparing the full model, including batch, bleed age, litter, pen, and genetic effects to reduced models without each litter or pen effect (Hagger, 1998; Gilmour et al., 2015).

The CBC phenotype data were analyzed using linear mixed effects models to estimate the least-squares means for CBC traits by group (RES, MID, SUS, and DEAD), and the Tukey–Kramer test was applied for pairwise comparisons of the difference between groups in R (R Core Team, 2017, packages ‘lme4’ and ‘lsmeans’). White blood cell traits were log_10_-transformed because of residual heterogeneity. In the mixed model, batch was fitted as a fixed effect to control and minimize the influence of the environmental changes among batches, group was also fitted as a fixed effect, and bleeding age was fitted as a covariate. Of note, for the changes of CBC between time points, bleeding age of Blood 1 was fitted for Δ13 and Δ14, and Blood 3 bleeding age was fitted for Δ34 since the 4-week interval between each blood sampling was the same for all animals. Random terms, including the litter and pen effects were fitted if significant (*p* < 0.05).

Heritabilities and genetic correlations of CBC traits with resilience traits were estimated in ASReml4.1 using pairwise bivariate models, with batch, bleed age, litter, and pen effects as described above for estimating the difference between resilience groups. Analyses for GFGR and TR included the fixed effect of batch for both traits, and random effects of litter and pen if significant (*p* < 0.05). Animal genetic effects were fitted using the genomic relationship matrix for 2593 animals, rather than the pedigree-based relationship matrix because the complete pedigree was unavailable due to the use of pooled semen in some batches. The genomic relationship matrix was constructed using ZZ/′2∑pi(1-pi), where *Z* contains centered genotypes codes and *p*_*i*_ is the minor allele frequency for locus *i* (VanRaden, 2008). The average estimate of corresponding pairwise bivariate analyses was reported as the heritability for each trait. In the bivariate models, batch was fitted as a fixed effect for both traits. The likelihood ratio test was applied to test the significance of estimates for heritabilities and genetic correlations in ASReml 4.1, where the log-likelihood of full models were compared to restricted models that constrained the genetic variance and the genetic covariance to zero, respectively (Gilmour et al., 2015).

The model used in ASReml 4.1 can be written as

$$\left[\begin{array}{c} y_{1}\\ y_{2} \end{array}\right]=\left[\begin{array}{cc} X_{1} & 0\\ 0 & X_{2} \end{array}\right]\,\left[\begin{array}{c} b_{1}\\ b_{2} \end{array}\right]+\left[\begin{array}{cc} Z_{1} & 0\\ 0 & Z_{2} \end{array}\right]\,\left[\begin{array}{c} g_{1}\\ g_{2} \end{array}\right]+\left[\begin{array}{cc} Z_{3} & 0\\ 0 & Z_{4} \end{array}\right]\,\left[\begin{array}{c} c_{1}\\ c_{2} \end{array}\right]+\left[\begin{array}{c} e_{1}\\ e_{2} \end{array}\right]$$

where **y_1_** and **y_2_** denote vectors of observations for traits 1 and 2; **X_1_** and **X_2_** are incidence matrices relating fixed effects to **y_1_** and **y_2_**, **b_1_** and **b_2_** are vectors of fixed effects for traits 1 and 2; **Z_1_** and **Z_2_** represent design matrices that associate observations of traits 1 and 2 to vectors of animal genetic effects **g_1_** and **g_2_**; **c_1_** and **c_2_** are vectors of random effects, including litter and pen effects when they were significant (*p* < 0.05); **Z_3_** and **Z_4_** are incidence matrices relating **y_1_** and **y_2_** to random effects **c_1_** and **c_2_**; **e_1_** and **e_2_** are vectors of unknown and random residuals for traits 1 and 2 (Miar et al., 2014a, b; Gilmour et al., 2015).

When random effects **c** and residuals errors **e** are uncorrelated, and identically distributed following a normal distribution, the (co-)variances of random effects are assumed to be

$$\mathrm{Var}\,\left[\begin{array}{c} g_{1}\\ g_{2}\\ c_{1}\\ c_{2}\\ e_{1}\\ e_{2} \end{array}\right]=\left[\begin{array}{cccccc} G\,\sigma_{g_{1}}^{2} & G\,\sigma_{g_{1}\,g_{2}} & 0 & 0 & 0 & 0\\ G\,\sigma_{g_{1}\,g_{2}} & G\,\sigma_{g_{2}}^{2} & 0 & 0 & 0 & 0\\ 0 & 0 & I\,\sigma_{c_{1}}^{2} & I\,\sigma_{c_{1}\,c_{2}} & 0 & 0\\ 0 & 0 & I\,\sigma_{c_{1}\,c_{2}} & I\,\sigma_{c_{2}}^{2} & 0 & 0\\ 0 & 0 & 0 & 0 & I\,\sigma_{e_{1}}^{2} & I\,\sigma_{e_{1}\,e_{2}}\\ 0 & 0 & 0 & 0 & I\,\sigma_{e_{1}\,e_{2}} & I\,\sigma_{e_{2}}^{2} \end{array}\right]$$

where ***G*** is the genomic relationship matrix, ***I*** is the identity matrix, $\sigma_{g}^{2}$ is the additive genetic variance, $\sigma_{c}^{2}$ is the random effect variance, and $\sigma_{e}^{2}$ is the residual variance. σ_*g*1*g*2_, σ_*c*_1_*c*_2__, and σ_*e*_1_*e*_2__ are covariances between two traits due to the additive genetic effects, common random effects, and residual effects, respectively. Heritability (h^2^) of a trait was estimated using variance components obtained from the bivariate analyses, and the average estimates of corresponding pairwise bivariate analyses were reported as the heritabilities:

$$h^{2}=\sigma_{g}^{2}/\left(\sigma_{g}^{2}+\sigma_{c}^{2}+\sigma_{e}^{2}\right)$$

and the genetic correlation (r_g_) between two traits was estimated as:

$$r_{g}=\sigma_{g\,1\,g\,2}/\sigma_{g\,1}\,\sigma_{g\,2}$$

## Results

### Descriptive Statistics for CBC Traits

Table 1 summarizes the descriptive statistics for the CBC data of 2593 genotyped animals after removing outliers. Most traits were recorded on all animals in Blood 1, but some samples for Blood 3 and Blood 4 were unavailable for animals that died prior to the sampling. Relevant random effects fitted in the models for CBC traits are presented in Table 2. The random effect of litter was fitted for GFGR, and pen effects in the test station late nursery and the grow-to-finish stage were fitted for TR.

**TABLE 1:** Descriptive statistics for complete blood count (CBC) traits in Blood 1, Blood 3, and Blood 4 after removing outliers, including the number of animals per trait (n), mean, standard deviation (SD), minimum (Min), and maximum (Max) values.

Traits^1^	Blood 1					Blood 3					Blood 4					Reference intervals^2^	
				
	*n*	Mean	*SD*	Min	Max	*n*	Mean	*SD*	Min	Max	*n*	Mean	*SD*	Min	Max	0 to 42 days	42 day to 2 years
WBC, 10^3^/μL	2222	11.47	3.67	5.64	28.21	2284	19.11	5.09	8.28	36.53	1802	21.92	6.15	9.23	43.01	9.62–25.20	11.35–28.90
NEU, 10^3^/μL	2375	4.76	2.38	1.33	14.71	2322	10.34	4.01	1.64	23.61	1808	9.95	4.65	2.48	28.37	2.35–11.90	2.00–10.40
LYM, 10^3^/μL	2425	5.61	1.85	2.39	12.65	2326	6.47	2.21	2.06	13.57	1840	9.82	3.11	3.67	21.09	4.02–12.50	5.30–17.90
MONO, 10^3^/μL	2440	0.32	0.21	0.04	1.23	2364	0.82	0.59	0.05	3.70	1890	1.01	0.74	0	4.06	0.05–2.30	0–3.70
EOS, 10^3^/μL	2474	0.47	0.40	0	2.61	2213	0.71	0.75	0.12	4.35	1807	0.60	0.48	0.12	3.01	0–0.50	0–1.30
BASO, 10^3^/μL	2096	0.13	0.23	0.02	1.69	2264	0.84	1.36	0.06	8.51	1798	0.33	0.32	0.05	2.09	NA^3^	NA
RBC, 10^6^/μL	2373	6.15	0.60	4.27	7.52	2242	5.79	0.67	3.82	7.55	1767	6.28	0.57	4.51	7.67	4.87–7.88	5.88–8.19
HGB, g/L	2434	116.45	13.46	73	148	2239	100.59	10.35	68	126	1730	104.95	9.71	69	125	80.8–119	112–147
HCT,%	2310	37.12	4.10	24	44	2228	32.81	3.63	22.10	41.80	1723	35.25	3.14	28	43	28.22–39.80	32.30–42.60
MCV, fL	2444	61.25	5.45	44.5	73.40	2339	57.02	3.59	49.60	69.50	1879	55.78	3.42	46.80	65.40	43.40–64.50	47.50–59.20
MCH, pg	2318	18.73	2.03	12.50	23.60	2153	17.52	1.26	14.70	21.80	1719	16.72	1.19	13.40	20.10	12.40–19.30	16.30–20.60
MCHC, g/L	2245	305.88	12.06	274	340	2150	307.40	15.77	268	366	1708	300.22	13.31	264	345	273–314	333–358
RDW,%	2473	21.97	4.02	15.80	39.90	2321	18.45	1.61	15.90	25.10	1873	18.61	1.40	15.60	23.10	NA	NA
PLT, 10^3^/μL	2457	285.13	177.18	0	949	2351	365.46	182.69	35	1062	1872	337.08	150.87	47	784	374.3–1080.8	118.9–522.9
MPV, fL	2435	14.63	3.35	8.30	26.20	2180	15.33	3.72	10.10	30.80	1849	13.57	2.01	9.30	20.50	NA	NA

*^1^WBC: total white blood cell concentration; NEU: neutrophil concentration; LYM: lymphocyte concentration; MONO: monocyte concentration; EOS: eosinophil concentration; BASO: basophil concentration; RBC: red blood cell concentration; HGB: hemoglobin concentration; HCT: hematocrit; MCV: mean corpuscular volume; MCH: mean corpuscular hemoglobin; MCHC: mean corpuscular hemoglobin concentration; RDW: red blood cell distribution width; PLT: platelet concentration; MPV: mean platelet volume. ^2^Suggested reference intervals for CBC traits of 0 to 42 days-old pigs and 42 days-old to 2 years-old pigs (Iowa State University’s Clinical Pathology Laboratory, 2011). ^3^Not applicable.*

**TABLE 2:** Random effects included in the models for the analyses of complete blood count (CBC) traits.

Traits^1^	Blood 1		Blood 3			Blood 4				Δ13^2^			Δ34^3^				Δ14^4^			
						
	Litter	Pen1^5^	Litter	Pen1	Pen2^6^	Litter	Pen1	Pen2	Pen3^7^	Litter	Pen1	Pen2	Litter	Pen1	Pen2	Pen3	Litter	Pen1	Pen2	Pen3
WBC	√^8^	NS^9^	√	NS	√	NS	NS	NS	√	√	NS	√	NS	NS	NS	√	NS	NS	NS	√
NEU	√	NS	√	NS	√	NS	NS	NS	√	√	NS	√	NS	NS	NS	√	NS	NS	NS	√
LYM	√	NS	√	NS	√	NS	NS	NS	√	√	NS	NS	NS	NS	NS	√	NS	NS	NS	√
MONO	√	NS	NS	NS	√	√	√	NS	NS	NS	NS	NS	√	NS	NS	√	√	NS	NS	NS
EOS	√	√	NS	√	NS	NS	NS	NS	NS	NS	NS	NS	NS	√	NS	NS	√	NS	NS	√
BASO	NS	√	NS	√	√	√	√	NS	√	NS	√	√	NS	√	√	NS	NS	NS	NS	√
RBC	√	√	√	√	√	NS	NS	NS	√	√	√	NS	NS	NS	NS	√	√	√	NS	NS
HGB	√	√	√	√	√	NS	NS	NS	√	√	√	√	NS	NS	NS	√	√	√	NS	√
HCT	√	√	√	√	√	NS	NS	NS	√	√	√	NS	NS	NS	NS	√	√	√	NS	√
MCV	√	√	√	NS	NS	√	NS	NS	√	√	√	NS	√	NS	NS	√	√	NS	NS	NS
MCH	√	√	√	NS	√	√	NS	NS	NS	√	√	NS	√	NS	NS	√	√	NS	NS	√
MCHC	√	√	√	√	NS	NS	NS	NS	√	√	√	NS	NS	NS	NS	√	√	√	NS	√
RDW	√	√	√	√	√	√	√	NS	√	√	√	NS	√	NS	NS	√	√	NS	NS	NS
PLT	√	√	NS	NS	√	√	√	NS	NS	√	√	NS	NS	NS	NS	√	√	√	NS	NS
MPV	√	√	NS	NS	√	NS	NS	NS	√	NS	√	NS	NS	√	NS	NS	NS	√	NS	NS

*^1^WBC: total white blood cell concentration; NEU: neutrophil concentration; LYM: lymphocyte concentration; MONO: monocyte concentration; EOS: eosinophil concentration; BASO: basophil concentration; RBC: red blood cell concentration; HGB: hemoglobin concentration; HCT: hematocrit; MCV: mean corpuscular volume; MCH: mean corpuscular hemoglobin; MCHC: mean corpuscular hemoglobin concentration; RDW: red blood cell distribution width; PLT: platelet concentration; MPV: mean platelet volume. ^2^The change of CBC traits from Blood 1 to Blood 3; ^3^The change of CBC traits from Blood 3 to Blood 4; ^4^The change of CBC traits from Blood 1 to Blood 4. ^5^The pen arrangement in the healthy quarantine unit; ^6^The pen arrangement in the test station late nursery; ^7^The pen arrangement in the test station grow-to-finish stage. ^8^Significant random effect that was included in the model. ^9^Not significant.*

### Group Differences in CBC Traits

#### White Blood Cell Traits

Results comparing the least-squares means of white blood cell traits in groups with different responses to the natural disease challenge are shown in Table 3. In Blood 1, no significant difference was found between groups for any of the white blood cell traits. However, in Blood 3, the RES group had a significantly higher LYM, and the LYM for the MID group was also significantly higher than for the DEAD group (*FDR* = 0.0003). In Blood 4, the RES and MID groups had significantly lower NEU levels than both the SUS and DEAD groups (*FDR* = 0.0002). For the count of LYM in Blood 4, the DEAD group was significantly lower than both the RES and MID groups (*FDR* = 0.0012).

**TABLE 3:** Least-squares means ± standard errors for white blood cell traits^1^ in Blood 1, 3, and 4 of animals from the resilient (RES), average (MID), susceptible (SUS), and dead (DEAD) groups.

Blood 1,10^3^/μL	RES	MID	SUS	DEAD	FDR-group^2^
log_10_ (WBC)	1.03 ± 0.01^a3^	1.04 ± 0.00^a^	1.04 ± 0.01^a^	1.03 ± 0.01^a^	0.55
log_10_ (NEU)	0.62 ± 0.01^a^	0.64 ± 0.01^a^	0.63 ± 0.01^a^	0.62 ± 0.01^a^	0.55
log_10_ (LYM)	0.71 ± 0.01^a^	0.73 ± 0.00^a^	0.73 ± 0.01^a^	0.72 ± 0.01^a^	0.29
log_10_ (MONO)	−0.61 ± 0.02^a^	−0.59 ± 0.01^a^	−0.58 ± 0.02^a^	−0.58 ± 0.01^a^	0.42
log_10_ (EOS)	−0.47 ± 0.02^a^	−0.49 ± 0.01^a^	−0.50 ± 0.02^a^	−0.50 ± 0.01^a^	0.84
log_10_ (BASO)	−1.15 ± 0.02^a^	−1.15 ± 0.01^a^	−1.14 ± 0.02^a^	−1.16 ± 0.01^a^	0.88
**Blood 3,10^3^/μL**	**RES**	**MID**	**SUS**	**DEAD**	**FDR-group**
log_10_ (WBC)	1.27 ± 0.01^a^	1.27 ± 0.00^a^	1.25 ± 0.01^a^	1.26 ± 0.01^a^	0.18
log_10_ (NEU)	0.97 ± 0.01^a^	0.98 ± 0.00^a^	0.97 ± 0.01^a^	0.98 ± 0.01^a^	0.56
log_10_ (LYM)	**0.82 ± 0.01^c4^**	**0.79 ± 0.00^b^**	**0.77 ± 0.01^ab^**	**0.75 ± 0.01^a^**	**<0.0001**
log_10_ (MONO)	−0.18 ± 0.02^a^	−0.21 ± 0.01^a^	−0.23 ± 0.02^a^	−0.22 ± 0.01^a^	0.27
log_10_ (EOS)	−0.32 ± 0.02^a^	−0.30 ± 0.01^a^	−0.33 ± 0.02^a^	−0.33 ± 0.01^a^	0.15
log_10_ (BASO)	−0.51 ± 0.02^a^	−0.51 ± 0.01^a^	−0.55 ± 0.02^a^	−0.49 ± 0.01^a^	0.15
**Blood 4,10^3^/μL**	**RES**	**MID**	**SUS**	**DEAD**	**FDR-group**
log_10_ (WBC)	1.31 ± 0.01^a^	1.32 ± 0.00^a^	1.34 ± 0.01^a^	1.34 ± 0.01^a^	0.21
log_10_ (NEU)	**0.93 ± 0.01^a^**	**0.95 ± 0.01^a^**	**1.02 ± 0.01^b^**	**1.03 ± 0.02^b^**	**<0.0001**
log_10_ (LYM)	**0.98 ± 0.01^b^**	**0.98 ± 0.00^b^**	**0.95 ± 0.01^ab^**	**0.92 ± 0.01^a^**	**0.0009**
log_10_ (MONO)	−0.16 ± 0.02^a^	−0.15 ± 0.01^a^	−0.14 ± 0.02^a^	−0.18 ± 0.03^a^	0.67
log_10_ (EOS)	−0.33 ± 0.01^a^	−0.33 ± 0.01^a^	−0.29 ± 0.02^a^	−0.30 ± 0.02^a^	0.46
log_10_ (BASO)	−0.61 ± 0.02^a^	−0.59 ± 0.01^a^	−0.57 ± 0.02^a^	−0.57 ± 0.02^a^	0.40

*^1^WBC: total white blood cell concentration; NEU: neutrophil concentration; LYM: lymphocyte concentration; MONO: monocyte concentration; EOS: eosinophil concentration; BASO: basophil concentration. ^2^FDR-group: adjusted *p*-values for the significant level of group effect using the Benjamini and Hochberg correction (FDR) in R to control false positives from multiple comparisons (R Core Team, 2017 Package ‘stats’). ^3^Values in a column suffixed with different letters are significantly different from each other at *FDR* < 0.05. ^4^Significant differences among RES, MID, SUS and DEAD groups are highlighted in bold (*FDR* < 0.05).*

Results comparing the least-squares means of changes in white blood cell traits between groups are summarized in Table 4. All white blood cell traits increased from Blood 1 to Blood 3 shown as positive Δ13. The increase of LYM was significantly higher for the RES group than for the other groups (*FDR* = 0.0002), but no significant difference was found among the MID, SUS and DEAD groups. Changes of white blood cell traits from Blood 3 to Blood 4 were not as dramatic as those from Blood 1 to Blood 3, except for LYM, which had a higher increase from Blood 3 to Blood 4 for all groups. The WBC, LYM, and MONO levels increased continuously for all groups based on positive Δ13 and Δ34, but EOS and BASO decreased from Blood 3 to Blood 4 based on negative Δ34. NEU showed a tendency to decrease in the RES and MID groups, which was opposite to the positive NEU in the SUS and DEAD groups for Δ34 (*FDR* < 0.0024). Additionally, a significant difference in NEU among groups was also identified for Δ14, which represents the overall change of NEU from Blood 1 to Blood 4. Δ14 for NEU were positive for all groups, but the SUS and DEAD groups had significantly higher increases in NEU than the RES and MID groups (*FDR* = 0.0002). Compared with Blood 1, which was collected in the quarantine unit, the other white blood cell traits, including WBC, LYM, MONO, EOS, and BASO, also increased significantly in Blood 4, although no significant differences based on Δ14 were found between groups.

**TABLE 4:** Least-squares means ± standard errors for changes of white blood cell traits^1^ between Blood 1, 3, and 4 of animals in the resilient (RES), average (MID), susceptible (SUS), and dead (DEAD) groups.

Δ13^2^, 10^3^/μL	RES	MID	SUS	DEAD	FDR-group^5^
WBC	8.39 ± 0.39^a6^	7.68 ± 0.17^a^	7.10 ± 0.40^a^	7.74 ± 0.29^a^	0.24
NEU	5.52 ± 0.27^a^	5.52 ± 0.11^a^	5.35 ± 0.29^a^	5.93 ± 0.20^a^	0.38
LYM	**1.69 ± 0.17^b7^**	**0.85 ± 0.07^a^**	**0.67 ± 0.17^a^**	**0.51 ± 0.12^a^**	**<0.0001**
MONO	0.56 ± 0.03^a^	0.49 ± 0.01^a^	0.42 ± 0.03^a^	0.47 ± 0.02^a^	0.08
EOS	0.23 ± 0.04^a^	0.26 ± 0.01^a^	0.18 ± 0.04^a^	0.24 ± 0.03^a^	0.38
BASO	0.85 ± 0.08^a^	0.66 ± 0.04^a^	0.63 ± 0.07^a^	0.79 ± 0.06^a^	0.08
**Δ34^3^, 10^3^/μL**	**RES**	**MID**	**SUS**	**DEAD**	**FDR-group**
WBC	2.13 ± 0.51^a^	2.89 ± 0.23^a^	4.06 ± 0.54^a^	3.91 ± 0.68^a^	0.08
NEU	−**0.60 ± 0.41^a^**	−**0.33 ± 0.17^a^**	**1.36 ± 0.40^b^**	**1.77 ± 0.51^b^**	**<0.0001**
LYM	3.08 ± 0.25^a^	3.41 ± 0.11^a^	3.24 ± 0.26^a^	2.78 ± 0.32^a^	0.32
MONO	0.16 ± 0.05^a^	0.20 ± 0.02^a^	0.24 ± 0.05^a^	0.13 ± 0.06^a^	0.65
EOS	−0.08 ± 0.04^a^	−0.13 ± 0.02^a^	−0.04 ± 0.04^a^	−0.03 ± 0.06^a^	0.16
BASO	−0.54 ± 0.06^a^	−0.45 ± 0.03^a^	−0.38 ± 0.06^a^	−0.40 ± 0.08^a^	0.42
**Δ14^4^, 10^3^/μL**	**RES**	**MID**	**SUS**	**DEAD**	**FDR-group**
WBC	10.39 ± 0.48^a^	10.75 ± 0.19^a^	11.60 ± 0.51^a^	11.73 ± 0.63^a^	0.27
NEU	**4.75 ± 0.35^a^**	**5.11 ± 0.14^a^**	**6.83 ± 0.36^b^**	**7.32 ± 0.45^b^**	**<0.0001**
LYM	4.63 ± 0.23^a^	4.37 ± 0.09^a^	4.06 ± 0.24^a^	3.56 ± 0.31^a^	0.08
MONO	0.72 ± 0.04^a^	0.71 ± 0.02^a^	0.66 ± 0.04^a^	0.61 ± 0.05^a^	0.37
EOS	0.12 ± 0.03^a^	0.13 ± 0.01^a^	0.22 ± 0.03^a^	0.19 ± 0.04^a^	0.12
BASO	0.15 ± 0.03^a^	0.20 ± 0.01^a^	0.24 ± 0.02^a^	0.23 ± 0.03^a^	0.12

*^1^WBC: total white blood cell concentration; NEU: neutrophil concentration; LYM: lymphocyte concentration; MONO: monocyte concentration; EOS: eosinophil concentration; BASO: basophil concentration. ^2^The change of complete blood count (CBC) traits from Blood 1 to Blood 3; ^3^The change of CBC traits from Blood 3 to Blood 4; ^4^The change of CBC traits from Blood 1 to Blood 4. ^5^FDR-group: adjusted *p*-values for the significant level of group effect using the Benjamini and Hochberg correction (FDR) in R to control false positives from multiple comparisons (R Core Team, 2017 Package ‘stats’). ^6^Values in a column suffixed with different letters are significantly different from each other at *FDR* < 0.05. ^7^Significant differences among RES, MID, SUS and DEAD groups are highlighted in bold (*FDR* < 0.05).*

#### Red Blood Cell and Platelet Traits

Results of comparing red blood cell and platelet traits in the RES, SUS, MID, and DEAD groups are summarized in Table 5. No significant differences were identified between groups for either red blood cell or platelet traits in Blood 1. However, for Blood 3, RDW and MPV were significantly higher in the DEAD group than in the RES and MID groups (*FDR* < 0.002). For Blood 4, several red blood cell traits showed significant differences between groups. Notably, HGB, HCT, and MCH were found to be significantly lower in the SUS and DEAD groups than in the RES and MID groups (*FDR* < 0.0005). Moreover, RBC was significantly higher in the RES and MID groups than in the SUS and DEAD groups (*FDR* = 0.0036), and MCV was significantly lower in the DEAD group than in the others. In contrast, RDW and MPV were found to be significantly higher in the DEAD group than in the RES in Blood 4.

**TABLE 5:** Least-squares means ± standard errors for red blood cell and platelet traits^1^ in Blood 1, 3, and 4 of animals in in the resilient (RES), average (MID), susceptible (SUS), and dead (DEAD) groups.

Blood 1	RES	MID	SUS	DEAD	FDR-group^2^
RBC, 10^6^/μL	6.18 ± 0.03^a3^	6.18 ± 0.01^a^	6.13 ± 0.03^a^	6.15 ± 0.02^a^	0.58
HGB, g/L	117.15 ± 0.71^a^	116.84 ± 0.31^a^	116.89 ± 0.72^a^	116.50 ± 0.50^a^	0.92
HCT,%	37.45 ± 0.23^a^	37.50 ± 0.10^a^	37.63 ± 0.23^a^	37.22 ± 0.16^a^	0.51
MCV, fL	61.31 ± 0.28^a^	61.36 ± 0.13^a^	62.07 ± 0.28^a^	61.39 ± 0.20^a^	0.20
MCH, pg	18.67 ± 0.11^a^	18.67 ± 0.05^a^	18.83 ± 0.11^a^	18.69 ± 0.08^a^	0.67
MCHC, g/L	306.86 ± 0.68^a^	305.74 ± 0.29^a^	305.70 ± 0.68^a^	305.11 ± 0.47^a^	0.32
RDW,%	21.94 ± 0.22^a^	21.77 ± 0.10^a^	21.91 ± 0.22^a^	22.12 ± 0.16^a^	0.32
PLT, 10^3^/μL	281.02 ± 10.26^a^	283.85 ± 4.29^a^	290.49 ± 10.42^a^	286.12 ± 7.10^a^	0.93
MPV, fL	14.57 ± 0.16^a^	14.70 ± 0.07^a^	14.98 ± 0.16^a^	14.79 ± 0.11^a^	0.40
**Blood 3**	**RES**	**MID**	**SUS**	**DEAD**	**FDR-group**
RBC, 10^6^/μL	5.81 ± 0.04^a^	5.77 ± 0.02^a^	5.75 ± 0.04^a^	5.79 ± 0.03^a^	0.74
HGB, g/L	101.66 ± 0.60^a^	101.03 ± 0.25^a^	100.63 ± 0.61^a^	101.32 ± 0.44^a^	0.68
HCT,%	32.92 ± 0.20^a^	32.87 ± 0.09^a^	32.85 ± 0.21^a^	32.71 ± 0.15^a^	0.87
MCV, fL	57.15 ± 0.20^a^	57.15 ± 0.08^a^	57.22 ± 0.20^a^	56.80 ± 0.14^a^	0.29
MCH, pg	17.51 ± 0.07^a^	17.54 ± 0.03^a^	17.52 ± 0.08^a^	17.49 ± 0.06^a^	0.93
MCHC, g/L	306.66 ± 0.95^a^	306.68 ± 0.60^a^	304.65 ± 0.97^a^	307.79 ± 0.79^b^	0.14
RDW,%	**18.26 ± 0.10^a4^**	**18.40 ± 0.0^a^**	**18.58 ± 0.10^ab^**	**18.72 ± 0.07^b^**	**0.0004**
PLT, 10^3^/μL	390.20 ± 11.04^a^	362.14 ± 11.10^a^	305.14 ± 0.81^a^	363.44 ± 8.12^a^	0.26
MPV, fL	**14.59 ± 0.16^a^**	**14.92 ± 0.06^a^**	**15.01 ± 0.16^ab^**	**15.51 ± 0.12^b^**	**<0.0001**
**Blood 4**	**RES**	**MID**	**SUS**	**DEAD**	**FDR-group**
RBC, 10^6^/μL	**6.36 ± 0.04^b^**	**6.32 ± 0.01^b^**	**6.16 ± 0.04^a^**	**6.22 ± 0.05^ab^**	**0.0009**
HGB, g/L	**106.52 ± 0.60^b^**	**105.16 ± 0.25^b^**	**100.78 ± 0.62^a^**	**100.94 ± 0.81^a^**	**<0.0001**
HCT,%	**35.54 ± 0.20^b^**	**35.21 ± 0.08^b^**	**34.15 ± 0.22^a^**	**34.05 ± 0.28^a^**	**<0.0001**
MCV, fL	**56.03 ± 0.20^b^**	**55.74 ± 0.08^b^**	**55.44 ± 0.20^b^**	**54.39 ± 0.26^a^**	**<0.0001**
MCH, pg	**16.89 ± 0.07^b^**	**16.78 ± 0.03^b^**	**16.57 ± 0.07^a^**	**16.37 ± 0.09^a^**	**<0.0001**
MCHC, g/L	301.73 ± 0.72^a^	301.17 ± 0.30^a^	299.45 ± 0.73^a^	299.97 ± 0.94^a^	0.15
RDW,%	**18.31 ± 0.09^a^**	**18.57 ± 0.04^b^**	**18.84 ± 0.09^c^**	**18.89 ± 0.12^bc^**	**0.0001**
PLT, 10^3^/μL	352.11 ± 9.84^a^	337.37 ± 4.02^a^	354.67 ± 10.25^a^	339.54 ± 13.13^a^	0.38
MPV, fL	**13.31 ± 0.11^a^**	**13.41 ± 0.04^a^**	**13.63 ± 0.11^ab^**	**14.12 ± 0.13^b^**	**<0.0001**

*^1^RBC: red blood cell concentration; HGB: hemoglobin concentration; HCT: hematocrit; MCV: mean corpuscular volume; MCH: mean corpuscular hemoglobin; MCHC: mean corpuscular hemoglobin concentration; RDW: red blood cell distribution width; PLT: platelet concentration; MPV: mean platelet volume. ^2^FDR-group: adjusted *p*-values for the significant level of group effect using the Benjamini and Hochberg correction (FDR) in R to control false positives from multiple comparisons (R Core Team, 2017 Package ‘stats’). ^3^Values in a column suffixed with different letters are significantly different from each other at *FDR* < 0.05. ^4^Significant differences among RES, MID, SUS and DEAD groups are highlighted in bold (*FDR* < 0.05).*

Table 6 summarizes the results of comparing the least-squares means of changes in red blood cell and platelet traits between groups. In contrast to the increase in white blood cell traits, all red blood cell traits decreased from Blood 1 to Blood 3, except for MCHC, which increased significantly in the DEAD group. Apart from MCHC, the drop for the other red blood cell traits from Blood 1 to Blood 3 did not show a tendency of being different between groups. The MPV in the SUS group was the only platelet trait that did not show a significantly positive Δ13 due to a relatively large standard error. Changes of platelet traits based on Δ13 did not show significant differences between groups. In contrast to the decreasing trend of red blood cell traits from Blood 1 to Blood 3, RBC and HCT increased significantly from Blood 3 to Blood 4 for all groups based on positive Δ34. Moreover, HGB also increased for both the RES and MID groups from Blood 3 to Blood 4, and Δ34 for HGB of these groups was significantly different from Δ34 for the SUS and DEAD groups (*FDR* = 0.0002), which were not found to be significantly different from zero. The MCV decreased continuously based on negative Δ34, and the DEAD group showed a more dramatic drop in MCV than the RES and MID groups (*FDR* = 0.0003). MCH and MCHC also kept decreasing based on negative Δ34, and the decrease of MCH for the DEAD group was significantly higher than for the RES and MID groups. Platelet traits also reduced from Blood 3 to Blood 4 for all groups, except for PLT in the SUS group, which did not show a significantly negative Δ34 due to a relatively large standard error.

**TABLE 6:** Least-squares means ± standard errors for changes of red blood cell and platelet traits^1^ between Blood1, Blood 3, and Blood 4 of animals in the resilient (RES), average (MID), susceptible (SUS), and dead (DEAD) groups.

Δ13^2^	RES	MID	SUS	DEAD	FDR-group^5^
RBC, 10^6^/μL	−0.43 ± 0.05^a6^	−0.44 ± 0.02^a^	−0.43 ± 0.05^a^	−0.33 ± 0.03^a^	0.16
HGB, g/L	−15.57 ± 0.93^a^	−15.77 ± 0.38^a^	−16.25 ± 0.96^a^	−14.72 ± 0.68^a^	0.59
HCT,%	−4.58 ± 0.31^a^	−4.68 ± 0.13^a^	−4.83 ± 0.32^a^	−4.59 ± 0.23^a^	0.93
MCV, fL	−4.11 ± 0.26^a^	−4.31 ± 0.10^a^	−4.74 ± 0.26^a^	−4.44 ± 0.18^a^	0.43
MCH, pg	−1.22 ± 0.10^a^	−1.20 ± 0.04^a^	−1.30 ± 0.10^a^	−1.17 ± 0.07^a^	0.81
MCHC, g/L	−**0.63 ± 1.00^a7^**	**0.91 ± 0.42^ab^**	−**0.92 ± 1.04^a^**	**3.13 ± 0.74^b^**	**0.01**
RDW,%	−3.58 ± 0.20^a^	−3.37 ± 0.08^a^	−3.32 ± 0.20^a^	−3.63 ± 0.14^a^	0.44
PLT, 10^3^/μL	105.93 ± 14.20^a^	76.20 ± 5.73^a^	67.59 ± 10.26^a^	67.59 ± 10.26^a^	0.29
MPV, fL	0.29 ± 0.22^a^	0.40 ± 0.09^a^	0.10 ± 0.22^a^	0.79 ± 0.16^a^	0.14
**Δ34^3^**	**RES**	**MID**	**SUS**	**DEAD**	**FDR-group**
RBC, 10^6^/μL	0.56 ± 0.05^a^	0.54 ± 0.02^a^	0.40 ± 0.05^a^	0.36 ± 0.06^a^	0.01
HGB, g/L	**6.23 ± 0.79^b^**	**4.32 ± 0.34^b^**	**0.59 ± 0.81^a^**	−**0.64 ± 1.03^a^**	**<0.0001**
HCT,%	**2.61 ± 0.27^b^**	**2.22 ± 0.12^b^**	**1.15 ± 0.29^a^**	**1.04 ± 0.38^a^**	**0.0002**
MCV, fL	−**1.01 ± 0.20^b^**	−**1.40 ± 0.08^b^**	−**1.84 ± 0.20^ab^**	−**2.55 ± 0.25^a^**	**<0.0001**
MCH, pg	−**0.59 ± 0.07^c^**	−**0.73 ± 0.03^bc^**	−**0.94 ± 0.08^ab^**	−**1.01 ± 0.10^a^**	**0.0007**
MCHC, g/L	−4.22 ± 1.06^a^	−4.57 ± 0.45^a^	−4.61 ± 1.10^a^	−3.74 ± 1.43^a^	0.94
RDW,%	0.06 ± 0.10^a^	0.22 ± 0.04^a^	0.30 ± 0.10^a^	0.15 ± 0.13^a^	0.46
PLT, 10^3^/μL	−43.81 ± 14.00^a^	−30.55 ± 5.90^a^	−2.77 ± 14.53^a^	−52.74 ± 18.46^a^	0.21
MPV, fL	−**1.34 ± 0.17^ab^**	−**1.55 ± 0.07^a^**	−**1.24 ± 0.17^ab^**	−**0.76 ± 0.22^b^**	**0.02**
**Δ14^4^**	**RES**	**MID**	**SUS**	**DEAD**	**FDR-group**
RBC, 10^6^/μL	0.18 ± 0.05^a^	0.16 ± 0.02^a^	0.05 ± 0.05^a^	0.09 ± 0.06^a^	0.22
HGB, g/L	−10.75 ± 0.98^a^	−11.76 ± 0.39^a^	−14.96 ± 1.04^a^	−13.42 ± 1.35^a^	0.06
HCT,%	−1.93 ± 0.32^a^	−2.22 ± 0.12^a^	−3.15 ± 0.35^a^	−2.96 ± 0.43^a^	0.06
MCV, fL	−5.38 ± 0.29^a^	−5.84 ± 0.11^a^	−6.70 ± 0.30^a^	−6.74 ± 0.39^a^	0.01
MCH, pg	−**1.81 ± 0.11^b^**	−**1.99 ± 0.05^ab^**	−**2.33 ± 0.12^a^**	−**2.20 ± 0.15^ab^**	**0.02**
MCHC, g/L	−5.53 ± 1.02^a^	−5.40 ± 0.44^a^	−7.01 ± 1.04^a^	−5.05 ± 1.27^a^	0.58
RDW,%	−3.36 ± 0.22^a^	−2.93 ± 0.09^a^	−2.84 ± 0.23^a^	−3.68 ± 0.29^a^	0.08
PLT, 10^3^/μL	69.91 ± 13.48^a^	56.42 ± 5.35^a^	60.74 ± 14.30^a^	22.48 ± 18.13^a^	0.32
MPV, fL	−1.12 ± 0.18^a^	−1.24 ± 0.07^a^	−1.15 ± 0.19^a^	−0.40 ± 0.25^a^	0.05

*^1^RBC: red blood cell concentration; HGB: hemoglobin concentration; HCT: hematocrit; MCV: mean corpuscular volume; MCH: mean corpuscular hemoglobin; MCHC: mean corpuscular hemoglobin concentration; RDW: red blood cell distribution width; PLT: platelet concentration; MPV: mean platelet volume. ^2^The change of complete blood count (CBC) traits from Blood 1 to Blood 3; ^3^The change of CBC traits from Blood 3 to Blood 4; ^4^The change of CBC traits from Blood 1 to Blood 4. ^5^FDR-group: adjusted *p*-values for the significant level of group effect using the Benjamini and Hochberg correction (FDR) in R to control false positives from multiple comparisons (R Core Team, 2017 Package ‘stats’). ^6^Values in a column suffixed with different letters are significantly different from each other at *FDR* < 0.05. ^7^Significant differences among RES, MID, SUS and DEAD groups are highlighted in bold (*FDR* < 0.05).*

Although several traits increased slightly from Blood 3 to Blood 4, for the overall changes from Blood 1 to Blood 4, all traits decreased significantly based on negative Δ14, except for RBC and PLT. Comparing Blood 4 to Blood 1, RBC increased slightly for the RES and MID groups, but it showed a tendency to return to the same level as in Blood 1 for the SUS and DEAD groups. PLT increased significantly from Blood 1 to Blood 4 for the RES, MID, and SUS groups, with no significant change identified for the DEAD group. MCHC was the only trait that showed a significant difference between groups for Δ14, which was lower in the SUS group than in the RES group (*FDR* = 0.04).

### Estimates of Heritability

The GFGR was estimated to be moderately heritable (0.15 ± 0.04), but the heritability estimate of TR was low (0.04 ± 0.01). Heritability estimates for CBC traits with standard errors are in Table 7. Most CBC traits were moderately heritable, with estimates ranging from 0.11 ± 0.03 to 0.27 ± 0.04. A few red blood cell traits showed moderate to high heritability estimates, ranging from 0.30 ± 0.04 to 0.53 ± 0.05, including RBC, MCV, and MCH in Blood 3 and 4. Estimates of heritability were low for some CBC traits, including BASO, HGB, and HCT in Blood 1, PLT in Blood 3 and Blood 4, RDW in Blood 4, and also for the changes of many CBC traits based on Δ13, Δ34, and Δ14. Genetic variances of several traits, especially MONO, and some changes of EOS, BASO, HCT, PLT, and MPV were not found to be significantly different from zero based on likelihood ratio tests, which compared full models to restricted models that constrained the genetic variance to zero in ASReml 4.1 (*p* > 0.05) (Gilmour et al., 2015).

**TABLE 7:** Estimates of heritability ± standard error for complete blood count (CBC) traits.

Traits^1^	Blood 1	Blood 3	Blood 4	Δ13^2^	Δ34^3^	Δ14^4^
WBC	**0.16 ± 0.04^5^**	**0.22 ± 0.04**	**0.19 ± 0.04**	**0.09 ± 0.04**	**0.14 ± 0.04**	**0.15 ± 0.04**
NEU	**0.18 ± 0.04**	**0.18 ± 0.04**	**0.13 ± 0.04**	**0.11 ± 0.04**	**0.11 ± 0.04**	**0.07 ± 0.04**
LYM	**0.21 ± 0.04**	**0.21 ± 0.04**	**0.30 ± 0.04**	**0.11 ± 0.04**	**0.20 ± 0.04**	**0.24 ± 0.04**
MONO	0.05 ± 0.03	**0.12 ± 0.03**	0.02 ± 0.03	**0.08 ± 0.03**	0.00 ± 0.00	0.05 ± 0.04
EOS	**0.22 ± 0.04**	**0.19 ± 0.04**	**0.27 ± 0.04**	**0.07 ± 0.03**	0.00 ± 0.03	**0.08 ± 0.04**
BASO	**0.08 ± 0.04**	**0.10 ± 0.03**	**0.13 ± 0.04**	**0.06 ± 0.04**	**0.06 ± 0.04**	0.06 ± 0.05
RBC	**0.27 ± 0.04**	**0.30 ± 0.04**	**0.34 ± 0.05**	**0.08 ± 0.04**	0.04 ± 0.04	**0.08 ± 0.05**
HGB	**0.08 ± 0.03**	**0.16 ± 0.04**	**0.28 ± 0.05**	**0.16 ± 0.04**	**0.11 ± 0.04**	**0.09 ± 0.05**
HCT	**0.09 ± 0.03**	**0.23 ± 0.04**	**0.23 ± 0.04**	0.04 ± 0.03	0.04 ± 0.04	**0.10 ± 0.05**
MCV	**0.19 ± 0.04**	**0.38 ± 0.04**	**0.46 ± 0.05**	**0.08 ± 0.03**	**0.22 ± 0.05**	0.06 ± 0.04
MCH	**0.18 ± 0.04**	**0.39 ± 0.04**	**0.53 ± 0.05**	**0.15 ± 0.04**	**0.13 ± 0.05**	0.06 ± 0.05
MCHC	**0.13 ± 0.04**	**0.25 ± 0.04**	**0.26 ± 0.05**	**0.17 ± 0.04**	**0.20 ± 0.05**	**0.07 ± 0.05**
RDW	**0.13 ± 0.03**	**0.14 ± 0.04**	**0.08 ± 0.04**	**0.14 ± 0.04**	**0.09 ± 0.04**	**0.18 ± 0.05**
PLT	**0.15 ± 0.03**	**0.07 ± 0.03**	**0.08 ± 0.04**	0.01 ± 0.03	0.00 ± 0.03	0.04 ± 0.03
MPV	**0.11 ± 0.03**	**0.19 ± 0.04**	**0.23 ± 0.04**	0.02 ± 0.03	**0.10 ± 0.04**	**0.08 ± 0.04**

*^1^WBC: total white blood cell concentration; NEU: neutrophil concentration; LYM: lymphocyte concentration; MONO: monocyte concentration; EOS: eosinophil concentration; BASO: basophil concentration; RBC: red blood cell concentration; HGB: hemoglobin concentration; HCT: hematocrit; MCV: mean corpuscular volume; MCH: mean corpuscular hemoglobin; MCHC: mean corpuscular hemoglobin concentration; RDW: red blood cell distribution width; PLT: platelet concentration; MPV: mean platelet volume. ^2^The change of CBC traits from Blood 1 to Blood 3; ^3^The change of CBC traits from Blood 3 to Blood 4; ^4^The change of CBC traits from Blood 1 to Blood 4. ^5^Significant estimates of genetic variances are highlighted in bold based on the likelihood ratio test by comparing full models to restricted models that constrained genetic variances to zero in ASReml 4.1 (*p* < 0.05).*

### Estimates of Genetic Correlations

GFGR and TR were estimated to be negatively correlated, with a genetic correlation of −0.50 ± 0.16. Estimates of genetic correlations for CBC traits that showed significant differences among groups (RES, MID, SUS, and DEAD) and the resilience traits of GFGR and TR are summarized in Table 8. LYM in Blood 3 and its change based on Δ13, which had the highest levels in the RES group, showed significantly negative genetic correlations with TR of −0.38 ± 0.18 and −0.46 ± 0.24, respectively. HCT based on Δ34, which was significantly higher in the RES and MID groups, showed a high negative genetic correlation with TR (−0.82 ± 0.47). NEU in Blood 4, RDW in Blood 4, and the change of NEU based on Δ14, which all had higher counts in the SUS and DEAD groups, showed significantly positive genetic correlations with TR. Genetic correlations between these CBC traits and GFGR showed a tendency of being opposite to the positive genetic correlations with TR but had relatively large standard errors. NEU based on Δ34, which was significantly positive in the SUS and DEAD groups but not significantly different from zero in the RES and MID groups, was estimated to have a negative genetic correlation with GFGR (−0.45 ± 0.21). TR showed a tendency to have a positive genetic correlation with the NEU based on Δ34 but had a large standard error (0.44 ± 0.26). For CBC traits from Blood 1, RDW was the only trait that showed a significantly positive genetic correlation with TR (0.41 ± 0.20), while none of the other CBC traits from Blood 1 showed significant correlations with TR or GFGR due to having low estimates and relatively high standard errors (Supplementary Table 1). Estimates of genetic correlations for CBC traits within Blood 1, Blood 3, and Blood 4 are summarized in Supplementary Table 2, while estimates of genetic correlations for each CBC trait between Blood 1, Blood 3, and Blood 4 are shown in Supplementary Table 3. Genetic correlations between Δ13, Δ34, and Δ14 were also estimated for each CBC trait and are summarized in Supplementary Table 4.

**TABLE 8:** Estimates of genetic correlations ± standard errors for complete blood count (CBC) traits that showed significant differences among groups with the resilience traits of grow-to-finish growth rate (GFGR) and treatment rate (TR).

Traits^1^	GFGR	TR
**Blood3**		
LYM	0.10 ± 0.18	−**0.38 ± 0.18^2^**
RDW	−0.07 ± 0.21	0.39 ± 0.22
MPV	0.09 ± 0.18	0.26 ± 0.18
**Blood4**		
NEU	−0.31 ± 0.20	**0.50 ± 0.23**
LYM	0.16 ± 0.15	−0.28 ± 0.16
RBC	0.15 ± 0.15	−0.08 ± 0.17
HGB	0.04 ± 0.16	−0.25 ± 0.18
HCT	0.10 ± 0.17	−0.33 ± 0.19
MCV	−0.08 ± 0.15	−0.16 ± 0.16
MCH	−0.03 ± 0.14	−0.21 ± 0.15
RDW	−0.12 ± 0.28	**0.89 ± 0.26**
MPV	0.09 ± 0.17	0.11 ± 0.19
**Δ13^3^**		
LYM	0.15 ± 0.23	−**0.46 ± 0.24**
MCHC	−0.25 ± 0.20	0.26 ± 0.21
**Δ34^4^**		
NEU	−**0.45 ± 0.21**	0.44 ± 0.26
RBC	−0.33 ± 0.45	−0.35 ± 0.43
HGB	0.01 ± 0.25	−0.32 ± 0.28
HCT	−0.29 ± 0.44	−**0.82 ± 0.47**
MCV	0.03 ± 0.19	0.02 ± 0.20
MCH	0.25 ± 0.25	0.14 ± 0.28
MPV	−0.15 ± 0.26	−0.27 ± 0.28
**Δ14^5^**		
NEU	−0.32 ± 0.26	**0.76 ± 0.29**
MCV	0.02 ± 0.33	−0.02 ± 0.35
MCH	0.00 ± 0.35	0.26 ± 0.36

*^1^NEU: neutrophil concentration; LYM: lymphocyte concentration; MONO: monocyte concentration; RBC: red blood cell concentration; HGB: hemoglobin concentration; HCT: hematocrit; MCV: mean corpuscular volume; MCH: mean corpuscular hemoglobin; MCHC: mean corpuscular hemoglobin concentration; RDW: red blood cell distribution width. ^2^Significant estimates of genetic correlations are highlighted in bold based on the likelihood ratio test by comparing full models to restricted models that constrained the genetic covariance to zero in ASReml 4.1 (*p* < 0.05). ^3^The change of CBC traits from Blood 1 to Blood 3; ^4^The change of CBC traits from Blood 3 to Blood 4; ^5^The change of CBC traits from Blood 1 to Blood 4.*

## Discussion

### CBC Traits and Disease Resilience

Hematopoiesis, including the establishment and maintenance of all circulating cellular blood components, relies on the proliferation and differentiation of hematopoietic stem cells (HSCs) (Orkin and Zon, 2008; Zaretsky et al., 2014). In response to disturbances of the hematopoietic equilibrium, such as infection, extensive proliferation and increased differentiation of HSCs are required to meet the higher demand of immune effector cells (Shahbazian et al., 2004; Singh et al., 2008; Johns et al., 2009; Yáñez et al., 2009; Baldridge et al., 2011; Boettcher and Manz, 2017). In the natural challenge model, our results showed that all white blood cell traits increased significantly from Blood 1 to Blood 4, although some traits, including NEU, EOS, and BASO, decreased from Blood 3 to Blood 4 (Tables 4, 5). According to the reference intervals, white blood cell traits have the tendency to increase slightly with age, except for NEU, which tends to decrease with age (Table 1) (Iowa State University’s Clinical Pathology Laboratory, 2011). Eze et al. (2011) indicated that white blood cell traits did not vary significantly between clinically healthy piglets and adults raised under an intensive management system. Therefore, the significant increases of all white blood cell traits observed here are likely to result from recruiting phagocytes (monocytes, neutrophils), immunocytes (lymphocytes), and granulocytes (neutrophils, eosinophils, and basophils) to drive immune responses at the early stage of infection (George-Gay and Parker, 2003; Mitre and Nutman, 2006; Rothenberg and Hogan, 2006; Porwit et al., 2011).

Notably, resilient pigs had significantly higher LYM for Blood 3 and based on Δ13 compared to the other three groups. Lymphocytes are mainly indicative of initiation and execution of the adaptive immune responses due to their essential and multiple roles in adaptive immunity (Figure 1B). Higher LYM in the blood of resilient pigs may indicate earlier and greater adaptive immune responses and increase the transport of lymphocytes to the infected tissues. Resilient pigs may be primed to orchestrate immune responses against a wide variety of pathogens more efficiently together with the higher concentrations of lymphocytes in infected tissues at the early stage of infection and, therefore, limiting the adverse effect caused by infectious challenges (Wilkie and Mallard, 1999; Badri and Wood, 2003; Zabriskie, 2009; Zhu et al., 2010; Luckheeram et al., 2012). This was also indicated by the negative genetic relationships of TR with LYM in Blood 3 and its change based on Δ13. A higher increase of LYM from Blood 1 to Blood 3 should favor resilience, which is related to a lower TR. Neutrophils, which increased significantly from Blood 1 to Blood 3 for all groups are both present as phagocytes and granulocytes in the innate immune response to defend against bacterial pathogens (Figure 1A) (Pham, 2006; Kolaczkowska and Kubes, 2013; Boettcher and Manz, 2017). However, after moving animals into the grow-to-finish stage, between Blood 3 and Blood 4, NEU showed the tendency to decrease in the RES and MID groups, which was opposite to the significant rise observed for the SUS and DEAD groups. Thus, NEU in Blood 4, and its changes based on Δ34 and Δ14 were also significantly lower for the RES and MID groups compared to the SUS and DEAD groups. Sustained high levels of NEU for the SUS and DEAD groups may be related to ongoing bacterial infection. The decrease of NEU in the blood of the RES and MID groups may indicate the recovery and resolution of inflammation when pathogens were brought under control by early initiation and efficient adaptive immune responses in resilient animals with higher increase of LYM from Blood 1 to Blood 3 (Savill, 1997; Nathan, 2006). Alternatively, it may reflect that neutrophils were already transported to the infected tissues to defend against pathogens in the RES and MID groups. These suggested processes need to be further explored for example, by monitoring the pathogen load in animals and identifying signs of the resolution of inflammation, such as the exodus of neutrophils in infected tissues and “stop signals” or checkpoints of inflammation, including lipoxins, Resolvins, and D-series prostaglandins (Serhan et al., 2007). Positive genetic correlations of TR with NEU in Blood 4 and its change based on Δ14, and the negative genetic correlation of GFGR with NEU based on Δ34 together may indicate that higher NEU in the grow-to-finish stage has a negative relationship with resilience, which is associated with increased TR and decreased GFGR.

Unlike the situation of white blood cells, red blood cell traits declined from Blood 1 to Blood 3 to the same degree for all groups, except for MCHC, which did not show a significant decrease (Table 6). By comparing clinically healthy grower to finisher pigs, Ježek et al. (2018) suggested that red blood cell traits, including RBC, HGB, HCT, MCV, and MCH, increased with age. The reference intervals from Iowa State University’s Clinical Pathology Laboratory (2011) also indicated a tendency for red blood cell traits to increase with age in pigs. Therefore, significant decreases in red blood cell traits from Blood 1 to Blood 3 are likely caused by the challenge of bacterial pathogens, which could damage circulating blood cells and accelerate hemolysis for iron to support bacterial cellular processes of respiration and replication (Barrett-Connor, 1972; Kent, 1994; Viana, 2011; Cassat and Skaar, 2013). This, however, changed during the late stage of infection for the RES and MID groups, for which HGB and HCT increased significantly from Blood 3 to Blood 4. Although red blood cell traits may increase with age, the significantly higher increase of HGB and HCT from Blood 3 to Blood 4 of more resilient animals may also suggest a better performance and faster recovery from infection by providing a higher level of iron and oxygen to the host (Morera and MacKenzie, 2011). Moreover, hemoglobin has been found to directly participate in immune responses as a source of bioactive peptides that exhibit antimicrobial activity against bacteria (El Bishlawy, 1999; Liepke et al., 2003). The higher increase of HGB from Blood 3 to Blood 4 of resilient animals are expected to enhance immune responses and work together with the other immune cells to defend against pathogens. Although relatively large standard errors are reported, highly negative genetic correlations of TR with HGB and HCT based on Δ34 and in Blood 4 may indicate that higher HGB and HCT during the late stage of infection favors resilience, which is related to lower TR. In addition, the significant increase in RDW has been identified to be a valuable index for assessing various pathological conditions, including inflammation and respiratory diseases in humans (Goyal et al., 2017). Our results also showed higher levels of RDW in Blood 3 and Blood 4 for less resilient animals. According to the highly positive genetic correlation of TR with RDW in Blood 4 (0.89 ± 0.26), higher RDW after challenge may have adverse effects associated with increasing the TR.

Significant genetic correlations of CBC traits with resilience traits suggest that a well-functioning immune system plays an essential role in resilient animals to maintain performance and prevent death from infection. An adequate nutritional status is necessary for the normal functioning of various components of the immune system because the immune system is energetically expensive (Coop and Kyriazakis, 2001; McDade, 2005; Nelson and Williams, 2007; Calder, 2013). Any changes in resource demands by the immune system can create significant differences in the level of fitness and performance that are related to resilience (Stearns, 1976). When nutrient resources are limited by decreased feed intake in response to disease challenge, a trade-off is expected to occur between the immune system and other nutrient-demands, such as growth (Lochmiller and Deerenberg, 2000; Doeschl-Wilson et al., 2009; Rauw, 2012; Putz et al., 2018). Although the negative genetic correlation between GFGR and TR could be the result of decreasing feed intake in challenged pigs, it might further indicate the trade-off and competing demands for the investment of nutrients in growth and immune function. In susceptible and dead animals, the infection may not be eliminated effectively as a result of a weak immune response. Therefore, decreased feed intake, along with prolonged infection, may further compromise the immune system, leading to a more severe disease state, and increased susceptibility to other pathogens (Keusch, 2003; Nelson and Williams, 2007; Hine et al., 2014). Conversely, the significant changes of CBC traits over time in RES animals, including higher LYM based on Δ13, higher HGB and HCT based on Δ34, and lower NEU based on Δ34 together, are expected to indicate the allocation of more resources toward immunity during the infection stage to help limit infection in resilient animals. Once the infection is brought under control by an efficient immune response, resilient animals may recover earlier from the infection, which could allow them to allocate more resources to maintain a higher growth rate in the grow-to-finish stage (McDade, 2005; Calder, 2013).

### Estimates of Heritabilities

Estimates of heritabilities for CBC traits have been reported in many studies (Table 9). Some of these were conducted under a controlled environment with limited disease challenges and types of pathogens (Clapperton et al., 2008, 2009). Others were conducted under a lower health status condition with multiple pathogens (Henryon et al., 2006; Flori et al., 2011; Mpetile et al., 2015). Heritability estimates for CBC traits in the natural challenge model in this study were within the range of estimates reported in these studies. Additionally, we were able to provide heritability estimates for novel CBC traits that capture changes of CBC in response to the challenge of infection. Heritability estimates for many CBC traits, especially red blood cells, were observed to be higher in Blood 3 and Blood 4 than in Blood 1, possibly because genetic variances of these traits may be more fully expressed in a lower health environment when there is the challenge of infection (Clapperton et al., 2008, 2009).

**TABLE 9:** Heritability estimates of complete blood count traits in related studies reported in the literature.

Traits^1^	Henryon et al., 2006	Clapperton et al., 2008				Clapperton et al., 2009		Flori et al., 2011	Mpetile et al., 2015
			
		SPF^2^	Non-SPF^3^	Start-test^4^	End-test^5^	SPF	Non-SPF		
WBC	0.25 ± 0.05	0.06 ± 0.11	0.37 ± 0.16	0.24 ± 0.15	0.18 ± 0.11	0.29 ± 0.13	0.28 ± 0.11	0.73 ± 0.20	0.23 ± 0.19
NEU	0.22 ± 0.04	−	−	−^6^	−	−	−	0.61 ± 0.20	0.31 ± 0.21
LYM	0.24 ± 0.05	−	−	−	−	−	−	0.72 ± 0.21	0.15 ± 0.19
MONO	0.22 ± 0.04	0.58 ± 0.18	0.58 ± 0.18	0.52 ± 0.17	0.59 ± 0.14	0.26 ± 0.11	0.16 ± 0.13	0.38 ± 0.20	0.36 ± 0.20
EOS	0.30 ± 0.05	−	−	−	−	−	−	0.80 ± 0.21	0.58 ± 0.12
BASO	−	−	−	−	−	−	−	−	0.12 ± 0.19
RBC	−	−	−	−	−	−	−	0.43 ± 0.20	0.62 ± 0.25
HGB	−	−	−	−	−	−	−	−	0.56 ± 0.13
HCT	−	−	−	−	−	−	−	−	0.06 ± 0.14
MCV	−	−	−	−	−	−	−	−	0.47 ± 0.24
RDW	−	−	−	−	−	−	−	0.70 ± 0.20	0.34 ± 0.25
MCH	−	−	−	−	−	−	−	−	0.37 ± 0.24
MCHC	−	−	−	−	−	−	−	−	0.04 ± 0.16
PLT	−	−	−	−	−	−	−	0.56 ± 0.19	0.11 ± 0.23
MPV	−	−	−	−	−	−	−	−	0.38 ± 0.25

*^1^WBC: total white blood cell concentration; NEU: neutrophil concentration, LYM: lymphocyte concentration; MONO: monocyte concentration; EOS: eosinophil concentration; BASO: basophil concentration; RBC: red blood cell concentration; HGB: hemoglobin concentration; HCT: hematocrit; MCV: mean corpuscular volume; RDW: red blood cell distribution width; MCH: mean corpuscular hemoglobin; MCHC: mean corpuscular hemoglobin concentration; PLT: platelet concentration; MPV: mean platelet volume. ^2^Specific pathogen-free (SPF), free of all major swine pathogens; ^3^Non-specific pathogen-free (Non-SPF), lower health status condition with the challenge of enzootic pneumonia, *Pasteurella multocida*, *Actinobacillus pleuropneumoniae*, *Leptospira Bratislava*, *Salmonella typhimurium*, and porcine multi-wasting syndrome; ^4^Blood samples collected from animals in both SPF and non-SPF farms at the average of 89 days old; ^5^Blood samples collected from animals in both SPF and non-SPF farms at the average of 148 days old; ^6^Heritability estimate of the trait was not reported in the study.*

Heritability estimates for GFGR and TR in this study were 0.15 ± 0.04 and 0.04 ± 0.01, respectively. Guy et al. (2018) estimated the heritability of treatments for a relatively high-health herd to be between 0.04 ± 0.03 and 0.06 ± 0.04. Putz et al. (2018) estimated the heritability of finishing average daily gain (FinADG) to be 0.25 ± 0.07 based on the phenotypes of the first three cycles of this natural challenge model. Moreover, the heritability for treatment rate adjusted to 180 days for animals that reached 65 days of age (TRT180) was estimated to 0.29 ± 0.07 by Putz et al. (2018). Our use of phenotypes and genotypes on a larger population with 2593 animals of six cycles resulted in relatively lower estimates of heritabilities and lower standard errors for both growth and treatment traits. Moreover, heritability estimates for the treatment rate were different since the definitions of this trait were not the same. In Putz et al. (2018), animals that died before the age of 65 days were excluded, but we included all animals unless they died without receiving any treatment. Moreover, we used additional batches of animals that were introduced into the natural challenge. As disease pressure varied by batch and on a seasonal basis, treatment rates could change accordingly. Moreover, treatment rates may also change with many other non-infectious factors, such as the level of stress caused by weather and transport in these batches (Bishop and Woolliams, 2014). Therefore, the heritability estimates for treatment rates are expected to change correspondingly.

## Conclusion

Resilience is a valuable attribute in livestock to manage infectious diseases and sustainably increase production efficiency, as resilient animals can maintain their performance without the need for intensive treatment. Consequently, there is an increasing focus on exploring the potential to select for resilience. Although CBC in Blood 1 is attractive as a potential predictor trait for resilience, as it is a cost-effective phenotype that can be collected from nucleus breeding herds with high health, no significant differences in CBC traits between resilience groups were identified for Blood 1 and estimates of genetic correlations of Blood 1 CBC traits with resilience were not significantly different from zero. Alternatively, for CBC under disease, resilient animals were found to have a greater increase of lymphocyte levels in the blood collected at 2-weeks after challenge, higher levels of hemoglobin and hematocrit, but a significantly lower level of the neutrophil concentration based on the changes from 2- to 6-weeks. Therefore, these changes of CBC traits in response to a disease challenge could provide a measure of resilience. Several of the latter CBC traits were found to be heritable and genetically correlated with resilience. Thus, these CBC traits may have the potential to be further developed as a phenotype for prediction of resilience by collecting data from commercial systems.

## Data Availability Statement

Because the data were generated on samples from commercially owned animals, the data analyzed in the current study are not publicly available, but they can be made available for non-commercial use by the corresponding author on reasonable request.

## Ethics Statement

The animal study was reviewed and approved by the Animal Protection Committee of the Centre de Recherche en Sciences Animales de Deschambault (15PO283) and the Animal Care and Use Committee at the University of Alberta (AUP00002227).

## Author Contributions

XB analyzed the data and wrote the manuscript with help from GP and ZW. FF, JH, PC, MD, JD, and GP designed the project and developed protocols for the natural disease challenge model. FF oversaw the sample collection and scheduling. JH was in charge of veterinary oversight on the project. CF provided support on CBC data measurement. GP was in charge of the database and genotyping for the project. AP and JD further processed the genotype data and provided the genomic relationship matrix for the project. All authors helped with the interpretation of results and reviewed and approved the final manuscript.

## Conflict of Interest

FF was employed by company Centre de Développement du Porc du Québec, Inc. The remaining authors declare that the research was conducted in the absence of any commercial or financial relationships that could be construed as a potential conflict of interest.

## Footnotes

1 https://www.ccac.ca/en/certification/about-certification
