# Supplementary material for: Exploring Phenotypes for Disease Resilience in Pigs Using Complete Blood Count Data From a Natural Disease Challenge Model
Source: Front Genet. 2020 Mar 13;11:216. doi: 10.3389/fgene.2020.00216 (PMC7083204; doi:10.3389/fgene.2020.00216)
Supplement: Supplementary file 1 [file Data_Sheet_1.docx]

Supplementary Material

**Supplementary Table 1.** Estimates of genetic correlations ± standard errors for complete blood count traits in Blood 1 with the resilience traits of grow-to-finish growth rate (GFGR) and treatment rate (TR).

| **Traits^1^** | **GFGR** | **TR** |
| --- | --- | --- |
| WBC | -0.09 ± 0.21 | -0.14 ± 0.22 |
| NEU | -0.14 ± 0.19 | -0.14 ± 0.20 |
| LYM | -0.05 ± 0.18 | 0.01 ± 0.19 |
| MONO | -0.50 ± 0.29 | 0.58 ± 0.31 |
| EOS | 0.01 ± 0.17 | 0.18 ± 0.18 |
| BASO | -0.07 ± 0.29 | 0.36 ± 0.31 |
| RBC | 0.16 ± 0.16 | 0.03 ± 0.17 |
| HGB | 0.09 ± 0.24 | 0.02 ± 0.26 |
| HCT | 0.05 ± 0.24 | -0.13 ± 0.25 |
| MCV | -0.23 ± 0.17 | -0.18 ± 0.18 |
| MCH | -0.08 ± 0.19 | -0.21 ± 0.19 |
| MCHC | 0.18 ± 0.21 | -0.25 ± 0.22 |
| RDW | -0.16 ± 0.20 | **0.41 ± 0.20^2^** |
| PLT | -0.16 ± 0.19 | -0.02 ± 0.21 |
| MPV | -0.18 ± 0.21 | 0.19 ± 0.22 |

^1^WBC: total white blood cell concentration; NEU: neutrophil concentration; LYM: lymphocyte concentration; MONO: monocyte concentration; EOS: eosinophil concentration; BASO: basophil concentration; RBC: red blood cell concentration; HGB: hemoglobin concentration; HCT: hematocrit; MCV: mean corpuscular volume; MCH: mean corpuscular hemoglobin; MCHC: mean corpuscular hemoglobin concentration; RDW: red blood cell distribution width; PLT: platelet concentration; MPV: mean platelet volume.

^2^Significant estimates of genetic correlations are highlighted in bold based on the likelihood ratio test by comparing full models to restricted models that constrained the genetic covariance to zero in ASReml 4.1 (*p < 0.05*).

**Supplementary Table 2.** Estimates of genetic correlations ± standard errors for complete blood count traits^1^ within Blood 1, Blood 3, and Blood 4.

| **Blood 1** | **WBC** | **NEU** | **LYM** | **MONO** | **EOS** | **BASO** | **RBC** | **PLT** |
| --- | --- | --- | --- | --- | --- | --- | --- | --- |
| WBC | - | Symmetric | | | | | | |
| NEU | **0.59 ± 0.09^2^** | - |  |  |  |  |  |  |
| LYM | **0.70 ± 0.09** | 0.24 ± 0.14 | - |  |  |  |  |  |
| MONO | **0.30 ± 0.12** | 0.28 ± 0.24 | **0.47 ± 0.18** | - |  |  |  |  |
| EOS | **0.42 ± 0.14** | **0.46 ± 0.12** | 0.24 ± 0.13 | 0.36 ± 0.21 | - |  |  |  |
| BASO | 0.23 ± 0.32 | **0.65 ± 0.16** | **0.70 ± 0.17** | **0.87 ± 0.27** | 0.31 ± 0.22 | - |  |  |
| RBC | 0.00 ± 0.15 | -0.07 ± 0.14 | -0.22 ± 0.13 | -0.44 ± 0.25 | 0.18 ± 0.12 | 0.19 ± 0.21 | - |  |
| PLT | **-0.42 ± 0.18** | -0.15 ± 0.17 | -0.16 ± 0.16 | -0.52 ± 0.31 | **-0.45 ± 0.13** | 0.05 ± 0.26 | 0.11 ± 0.14 | - |
| **Blood 3** | **WBC** | **NEU** | **LYM** | **MONO** | **EOS** | **BASO** | **RBC** | **PLT** |
| WBC | - | Symmetric | | | | | | |
| NEU | **0.83 ± 0.05** | - |  |  |  |  |  |  |
| LYM | **0.76 ± 0.08** | **0.36 ± 0.15** | - |  |  |  |  |  |
| MONO | **0.65 ± 0.12** | **0.34 ± 0.16** | **0.63 ± 0.12** | - |  |  |  |  |
| EOS | **0.41 ± 0.12** | 0.25 ± 0.15 | 0.22 ± 0.14 | 0.21 ± 0.17 | - |  |  |  |
| BASO | **0.57 ± 0.13** | **0.53 ± 0.14** | 0.23 ± 0.18 | **0.43 ± 0.19** | -0.05 ± 0.19 | - |  |  |
| RBC | 0.16 ± 0.12 | 0.17 ± 0.14 | 0.18 ± 0.13 | -0.08 ± 0.14 | -0.14 ± 0.13 | -0.13 ± 0.16 | - |  |
| PLT | -0.08 ± 0.21 | -0.11 ± 0.23 | -0.24 ± 0.22 | -0.12 ± 0.25 | 0.10 ± 0.21 | 0.09 ± 0.26 | -0.15 ± 0.17 | - |
| **Blood 4** | **WBC** | **NEU** | **LYM** | **MONO** | **EOS** | **BASO** | **RBC** | **PLT** |
| WBC | - | Symmetric | | | | | | |
| NEU | **0.79 ± 0.08** | - |  |  |  |  |  |  |
| LYM | **0.82 ± 0.07** | 0.25 ± 0.16 | - |  |  |  |  |  |
| MONO | **1.23 ± 0.35** | **1.03 ± 0.44** | 0.95 ± 0.48 | - |  |  |  |  |
| EOS | **0.45 ± 0.12** | **0.52 ± 0.14** | 0.01 ± 0.12 | 0.56 ± 0.46 | - |  |  |  |
| BASO | **0.93 ± 0.09** | **0.77 ± 0.14** | **0.76 ± 0.15** | 1.18 ± 0.65 | **0.50 ± 0.14** | - |  |  |
| RBC | **0.34 ± 0.14** | 0.20 ± 0.16 | **0.35 ± 0.11** | 0.03 ± 0.24 | -0.15 ± 0.12 | 0.13 ± 0.17 | - |  |
| PLT | -0.08 ± 0.26 | 0.00 ± 0.29 | 0.01 ± 0.20 | -0.45 ± 1.04 | **-0.15 ± 0.03** | 0.10 ± 0.30 | 0.03 ± 0.19 | - |

^1^WBC: total white blood cell concentration; NEU: neutrophil concentration; LYM: lymphocyte concentration; MONO: monocyte concentration; EOS: eosinophil concentration; BASO: basophil concentration; RBC: red blood cell concentration; PLT: platelet concentration.

^2^Significant estimates of genetic correlations are highlighted in bold based on the likelihood ratio test by comparing full models to restricted models that constrained the genetic covariance to zero in ASReml 4.1 (*p < 0.05*).

**Supplementary Table 3.** Estimates of genetic correlations ± standard errors for each complete blood count trait between Blood 1, Blood 3, and Blood4.

| **Traits^1^** | **Blood 1 & Blood 3** | **Blood 3 & Blood 4** | **Blood 1 & Blood 4** |
| --- | --- | --- | --- |
| WBC | **0.85 ± 0.13^2^** | **0.65 ± 0.12** | **0.62 ± 0.16** |
| NEU | **0.73 ± 0.12** | **0.80 ± 0.17** | **0.64 ± 0.16** |
| LYM | **0.68 ± 0.12** | **0.46 ± 0.12** | **0.57 ± 0.11** |
| MONO | **0.93 ± 0.25** | 1.26 ± 0.93 | 0.88 ± 0.89 |
| EOS | **0.40 ± 0.12** | **0.86 ± 0.12** | **0.60 ± 0.12** |
| BASO | **0.70 ± 0.28** | **0.49 ± 0.21** | **0.98 ± 0.29** |
| RBC | **0.86 ± 0.08** | **0.87 ± 0.07** | **0.82 ± 0.09** |
| HGB | **0.73 ± 0.23** | **0.79 ± 0.11** | **0.80 ± 0.19** |
| HCT | **0.60 ± 0.20** | **0.84 ± 0.11** | **0.69 ± 0.21** |
| MCV | **0.84 ± 0.06** | **0.81 ± 0.05** | **0.77 ± 0.08** |
| MCH | **0.77 ± 0.07** | **0.91 ± 0.04** | **0.79 ± 0.08** |
| MCHC | **0.49 ± 0.14** | **0.74 ± 0.11** | **0.74 ± 0.13** |
| RDW | **0.57 ± 0.13** | **0.62 ± 0.18** | -0.28 ± 0.31 |
| PLT | **0.78 ± 0.19** | **0.87 ± 0.26** | **0.99 ± 0.18** |
| MPV | **1.02 ± 0.16** | **0.76 ± 0.11** | **0.66 ± 0.14** |

^1^WBC: total white blood cell concentration; NEU: neutrophil concentration; LYM: lymphocyte concentration; MONO: monocyte concentration; EOS: eosinophil concentration; BASO: basophil concentration; RBC: red blood cell concentration; HGB: hemoglobin concentration; HCT: hematocrit; MCV: mean corpuscular volume; MCH: mean corpuscular hemoglobin; MCHC: mean corpuscular hemoglobin concentration; RDW: red blood cell distribution width; PLT: platelet concentration; MPV: mean platelet volume.

^2^Significant estimates of genetic correlations are highlighted in bold based on the likelihood ratio test by comparing full models to restricted models that constrained the genetic covariance to zero in ASReml 4.1 (*p < 0.05*).

**Supplementary Table 4.** Estimates of genetic correlations ± standard errors for the changes of each complete blood count (CBC) trait between Blood 1, Blood 3, and Blood 4.

| **Traits^1^** | **Δ13^2^ & Δ34^3^** | **Δ13 & Δ14^4^** | **Δ34 & Δ14** |
| --- | --- | --- | --- |
| WBC | **-0.45 ± 0.21^5^** | 0.36 ± 0.23 | **0.67 ± 0.13** |
| NEU | -0.39 ± 0.23 | 0.52 ± 0.28 | **0.62 ± 0.19** |
| LYM | **-0.52 ± 0.15** | 0.25 ± 0.17 | **0.76 ± 0.07** |
| MONO | -0.89 ± 0.71 | 0.48 ± 0.38 | 0.54 ± 0.72 |
| EOS | -0.42 ± 0.38 | **0.76 ± 0.23** | 0.95 ± 0.60 |
| BASO | **-0.92 ± 0.11** | -0.70 ± 0.76 | 0.42 ± 0.60 |
| RBC | **-0.76 ± 0.25** | 0.58 ± 0.42 | 0.25 ± 0.46 |
| HGB | -0.30 ± 0.19 | 0.32 ± 0.26 | 0.44 ± 0.25 |
| HCT | -0.93 ± 0.51 | 0.53 ± 0.42 | -0.15 ± 0.60 |
| MCV | -0.37 ± 0.22 | 0.44 ± 0.24 | **0.54 ± 0.22** |
| MCH | **-0.58 ± 0.25** | 0.02 ± 0.37 | 0.05 ± 0.35 |
| MCHC | **-0.76 ± 0.12** | **0.44 ± 0.22** | 0.36 ± 0.24 |
| RDW | **0.64 ± 0.32** | **0.98 ± 0.03** | **0.87 ± 0.16** |
| PLT | -0.44 ± 1.07 | 0.82 ± 1.03 | 0.62 ± 1.85 |
| MPV | **-0.77 ± 0.33** | 0.25 ± 0.50 | 0.34 ± 0.29 |

^1^WBC: total white blood cell concentration; NEU: neutrophil concentration; LYM: lymphocyte concentration; MONO: monocyte concentration; EOS: eosinophil concentration; BASO: basophil concentration; RBC: red blood cell concentration; HGB: hemoglobin concentration; HCT: hematocrit; MCV: mean corpuscular volume; MCH: mean corpuscular hemoglobin; MCHC: mean corpuscular hemoglobin concentration; RDW: red blood cell distribution width; PLT: platelet concentration; MPV: mean platelet volume.

^2^The change of CBC traits from Blood 1 to Blood 3; ^3^The change of CBC traits from Blood 3 to Blood 4; ^4^The change of CBC traits from Blood 1 to Blood 4.

^5^Significant estimates of genetic correlations are highlighted in bold based on the likelihood ratio test by comparing full models to restricted models that constrained the genetic covariance to zero in ASReml 4.1 (*p < 0.05*).
